# Evaluating feasibility of an automated 3-dimensional scanner using Raman spectroscopy for intraoperative breast margin assessment

**DOI:** 10.1038/s41598-017-13237-y

**Published:** 2017-10-19

**Authors:** G. Thomas, T.-Q. Nguyen, I. J. Pence, B. Caldwell, M. E. O’Connor, J. Giltnane, M. E. Sanders, A. Grau, I. Meszoely, M. Hooks, M. C. Kelley, A. Mahadevan-Jansen

**Affiliations:** 1 Vanderbilt Biophotonics Center, Vanderbilt University, Nashville, TN 37235 USA; 2 Department of Biomedical Engineering, Vanderbilt University, Nashville, TN 37235 USA; 3 Department of Biomedical Engineering, Northwestern University, Evanston, IL 60208 USA; 4 Genentech, San Francisco, CA 94080 USA; 5 Division of Pathology, Vanderbilt University Medical Center, Nashville, TN 37232 USA; 6 Division of Surgical Oncology, Vanderbilt University Medical Center, Nashville, TN 37232 USA

## Abstract

Breast conserving surgery is the preferred treatment for women diagnosed with early stage invasive breast cancer. To ensure successful breast conserving surgeries, efficient tumour margin resection is required for minimizing tumour recurrence. Currently surgeons rely on touch preparation cytology or frozen section analysis to assess tumour margin status intraoperatively. These techniques have suboptimal accuracy and are time-consuming. Tumour margin status is eventually confirmed using postoperative histopathology that takes several days. Thus, there is a need for a real-time, accurate, automated guidance tool that can be used during tumour resection intraoperatively to assure complete tumour removal in a single procedure. In this paper, we evaluate feasibility of a 3-dimensional scanner that relies on Raman Spectroscopy to assess the entire margins of a resected specimen within clinically feasible time. We initially tested this device on a phantom sample that simulated positive tumour margins. This device first scans the margins of the sample and then depicts the margin status in relation to an automatically reconstructed image of the phantom sample. The device was further investigated on breast tissues excised from prophylactic mastectomy specimens. Our findings demonstrate immense potential of this device for automated breast tumour margin assessment to minimise repeat invasive surgeries.

## Introduction

In 2017, an estimated 255,180 new cases of invasive breast cancer will be diagnosed in the American population, along with additional cases of 63,410 cases of *in-situ* lesions of the breast in women^1^. Of these cases, approximately 57% of the patients opt for breast conserving surgeries (BCS) over total mastectomy (complete removal of breast tissue)^2^. This is mainly because various studies have demonstrated that BCS has comparable patient outcomes with total mastectomy and yet provide superior cosmetic results^3,4^. As compared to total mastectomy, BCS retain most of the normal breast tissue and only remove the primary breast tumour with a surrounding margin of normal tissue. A clear margin devoid of tumour cells is classified as a ‘negative’ margin, whereas a margin with tumour cell infiltration is defined as a ‘positive’ margin. Since a positive margin is strongly associated with an increased risk for local tumour recurrence^5^, a surgeon always tries to ensure negative margins for successful BCS. However, the width of tumour margins chosen during surgeries ranges from < 1 mm to >1 cm depending on the surgeon, hospital, or anatomical and physiological constraints^6,7^. As a result, it has been challenging to set universal standards for defining an optimal negative margin for BCS^8,9^. While tumour margin width has been highly debated by surgeons, current consensus for invasive BC tumour surgeries is that no ink on tumour indicates a negative margin^8^. In comparison, a more stringent approach is recommended for ductal carcinoma *in situ* (DCIS) lesions that would require BCS along with whole breast radiation, where negative margin of at least 2 mm should be ensured^10^. This implies that surgeons need to ensure adequate resection for both invasive breast carcinoma and DCIS patients who opt for BCS. In terms of current intraoperative methods, margin status is assessed inside the operating room (OR) using simple visual examination, touch preparation cytology or frozen section analysis. On the other hand, all these techniques have significant drawbacks in terms of accuracy and time required^11–13^. Definitive margin status relies on post-operative histopathology as the gold standard. This information is however not available to the surgeon at the time of surgery. Consequently one in every 4–5 patients are recalled for invasive repeat surgeries due to insufficient tumour margin resection resulting from a lack of real-time feedback on margin status^14,15^. This can subject patients to additional discomfort, psychological stress, surgical complications, increased health care expenses and below-par cosmetic outcome^16^. Therefore surgeons essentially require an intraoperative tool that can detect positive margins in real-time to minimise repeat invasive surgeries.

At present, the only Food and Drug Administration (FDA) approved device available for automated intraoperative margin evaluation is the MarginProbe, which is based on differences in electromagnetic wave reflection between cancerous and normal tissue. According to a recent study, while the MarginProbe provided a sensitivity of 75%, it had a low specificity of 46% leading to high false positive rates^17^. Tumour margin assessment tools should preferably be: (i) highly accurate, (ii) able to scan entire specimen surface, (iii) non-interfering with the surgery, (iv) rapid (within 10 minutes), (v) sensitive for varying margin widths ≤ 2 mm^10^, (vi) operable on all specimens with varying shape, size and firmness and (vii) precisely co-register positive margins in excised specimen with the corresponding *in vivo* site in patient for immediate resection by surgeon. In addition to these properties, it would be the most ideal if the device for margin assessment was also automated. Designing an automated intraoperative tool for margin assessment could ensure reproducibility of margin assessment, minimize human error/variability from manual measurements, accurately co-register between spectral measurement point and corresponding site on specimen morphology, and most importantly reduce workload on the surgical personnel during BCS. Optical imaging methods can satisfy these requirements and obtain real-time tissue information in a non-invasive manner. Various studies have applied different optical methods for margin assessment, including the use of elastic scattering spectroscopy (ESS)^18^, diffuse reflectance spectroscopy (DRS)^19–21^, optical coherence tomography (OCT)^22^ and photoacoustic tomography (PAT)^23^.

Compared to these optical methods, Raman Spectroscopy (RS) has been demonstrated to be sensitive to subtle changes in tissue biochemistry^24^ that can be suitably tapped for sensitive margin assessment. Raman Spectroscopy (RS) involves measuring inelastically scattered light, where the acquired frequency shift of the detected light is related to the unique vibrational modes of the sample. Therefore each biological molecule has a distinct Raman spectrum, enabling RS to sensitively determine tissue biochemical composition. As a result, RS has been successfully exploited for breast cancer diagnostics in various studies with high sensitivity and specificity^25–30^. The discriminatory potential of RS was further explored for breast tumour margin assessment by Haka *et al*.^31^. The study demonstrated an overall accuracy of 93.3%, for detecting carcinoma from *in vivo* measurements conducted on the breast tumour bed. Spatially offset Raman spectroscopy (SORS), an advanced variant of RS, has recently been shown to obtain biological Raman spectra from greater tissue depths by aligning detector (D) fibres at varying offsets or distances from the source (S) fibre in the probe^32–34^. Detector fibres positioned further away from the source fibre are essentially more sensitive to photons travelling deeper beneath the tissue surface due to multiple scattering.

Keller *et al*. had earlier demonstrated the feasibility of SORS by adding offsets to detect depth resolved differences between soft tissues at submillimeter resolution^34^, which was followed by validation with Monte Carlo simulations^35^. The simulations indicated that a source-detector (S-D) offset less than 3.5 mm is required for the SORS probe to be sensitive up to a 2 mm depth in breast tissue^36,37^, to ensure adequate negative margin width^7,10,38^. A 5 mm diameter SORS probe using the optimised S-D offset was later developed, tested and validated *ex vivo* on frozen breast tissue samples, with a sensitivity of 95% and specificity of 100%^37^. However, the device was limited to performing single point measurements and would require numerous sequential spot measurements to evaluate the entire tumour surface, making the procedure time-consuming and laborious for this application.

While manual point-based breast margin assessment has been demonstrated with varying success rates in several studies^18,28^, there has been only limited research invested in evaluating the margin status of breast specimens in its entirety^19–22^. Additionally, tissue margin status expressed in relation to the gross morphology of the excised specimen in its entirety is more valuable for intraoperative surgical guidance. In this new paper, we describe a device, designated as ‘Marginbot’, which allows automated scanning of an excised specimen surface in 3D for margin evaluation. The goal of this study is to translate a point based assessment device to an automated 3D scanning instrument that could scan an entire lump within 10 minutes for intraoperative use. We accomplished this by extrapolating the point measurement approach described by Keller *et al*.^35^ to rapidly evaluate measure entire surface of resected specimens with minimal human intervention. For this purpose, we designed a new optical probe that could measure a larger surface area per measurement point, as compared to the probe tested by Keller *et al*.^35^. While the newly designed optical probe can acquire depth-resolved biochemical information for SORS, Raman spectra was obtained in a depth-averaged manner with the probe to ensure rapid spectral analysis of the margins within a clinically feasible duration. The prototype 3D scanner was then developed for automated scanning of the entire specimen surface utilising the new probe. Subsequently, software was customised to perform reliable and quick data processing and analysis for margin assessment. Eventually, the user-interface displays the margin analysis results superimposed on a 3D morphological image of the scanned specimen that could potentially guide surgeons towards the actual site requiring re-excision *in-situ*. We initially validated the performance of this system with a phantom sample that mimicked positive margins on breast tumour specimens. After optimising measurement sensitivity and scan speed, we tested the feasibility of the automated 3D scanner on breast tissue specimens obtained from prophylactic total mastectomies and validated the data with histopathological assessment.

## Results

### Developing and designing the device

**Figure 1:**
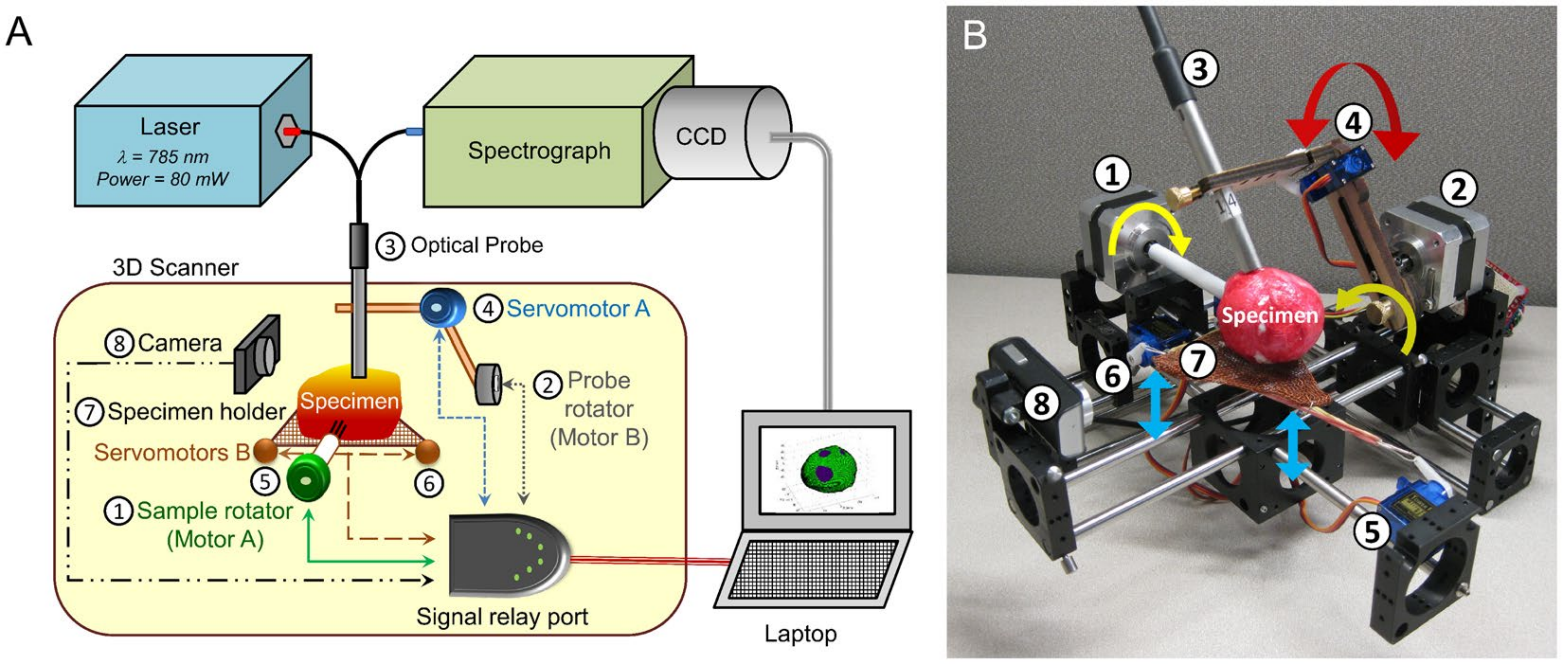
Design of the automated 3D margin scanner prototype (Marginbot). (**A**) Schematic of the system, (**B**) Photograph depicting arrangement of the various components used to scan specimen surface in 3D. The number coding in Fig. 1B is explained as follows. Motor A (1) rotates the specimen in the horizontal axis, while motor B (2) moves the optical probe (3) along the specimen surface. Servomotor A (4) enables contact mode and non-contact mode of the optical probe (1) with the specimen placed on the specimen holder (7). Servomotors B (5, 6) move the specimen holder (7) upwards pushing the specimen towards the probe during the contact mode and downwards in the non-contact mode. The compact camera (8) enables image reconstruction of the specimen being assessed for margin analysis.

The device essentially comprised of a 785 nm laser source, a spectrograph with a charge coupled detector (CCD), the newly designed optical Raman probe, a motorised 3D scanner (see Fig. 1) and a laptop for controlling the scanner and data processing. The 3D scanner enables probe movement over the sample surface during measurements.

For automated margin assessment of specimens in 3D, the scanner consisting of two motors, two servomotors, a specimen holder and a webcam camera was built as seen in Fig. 1A and B. Motor A rotates the specimen around its horizontal axis and motor B moves the optical probe along the specimen surface (as indicated in the arrow directions of Fig. 1B). For steady translocation of the probe from one measurement site to the next, the probe is stabilised by a probe-holder. Servomotor A moves the probe-holder up and down, leading the probe to come in contact mode with specimen during measurement and away from the specimen in non-contact mode after measurement. The mobile specimen holder moves up and down by activation of servomotors B, and is in contact with the specimen only during measurement. As a result, the specimen holder supports and prevents the specimen from sagging during measurements.

**Figure 2:**
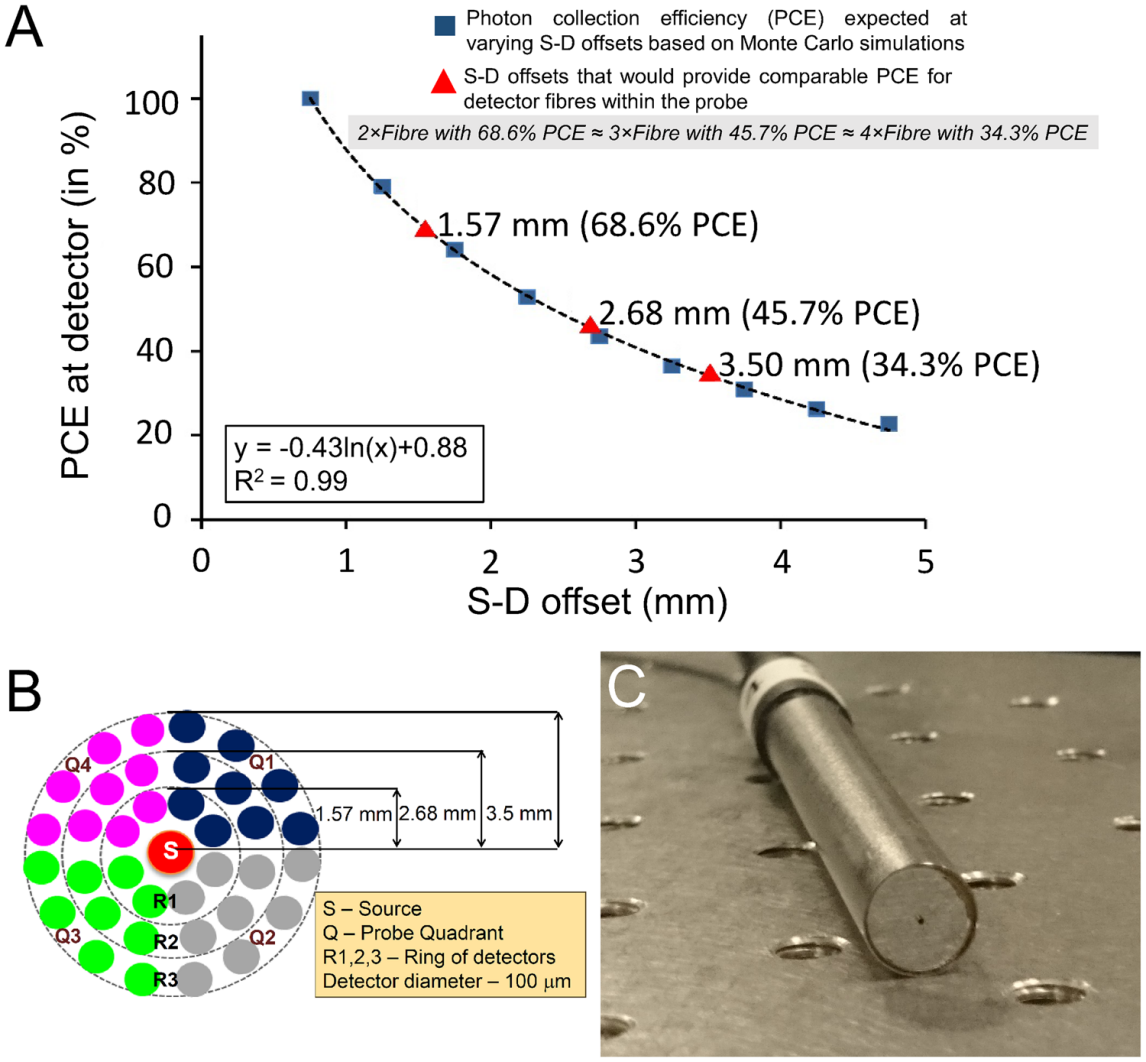
Design of the custom designed optical probe with potential for SORS. (**A**) Plot showing photon collection efficiency (PCE) at various source-detector (S-D) offsets based on Monte Carlo simulations (represented by blue rectangles). The red triangles represent S-D offsets (1.57, 2.68 and 3.5 mm) that allowed 68.6%, 45.7% and 34.3% PCE at R1, R2 and R3, relative to 100% at an S-D offset of 0.75 mm. At these S-D offsets, comparable PCE is obtained between the 3 ring of detectors by having 2, 3 and 4 detector fibres in R1, R2 and R3 respectively (2 fibres × 68.6% PCE ≈ 3 fibres × 45.7% PCE ≈ 4 fibres × 34.3% PCE). (**B**) Design of the probe tip. 36 detector fibres are divided into 4 quadrants (Q1-4) and three rings (R1-3) of detectors. (**C**) Photograph of the designed 7 mm diameter optical probe.

The probe is designed with the potential to acquire depth-resolved spectra for SORS. This was achieved by implementing S-D offsets for the custom detector rings using Monte Carlo (MC) simulations. The MC simulations aided in determining photon collection efficiency (PCE) at varying S-D offsets. As seen in Fig. 2A, the rectangular dots represent the PCE determined by MC simulations for varying S-D offsets and the curve represented the logarithm fitting. The primary consideration for probe design was to ensure comparable PCE between the detector fibres at various offsets. The triangles in Fig. 2A represent S-D offsets (1.57, 2.68 and 3.50 mm) that received a PCE of 68.6%, 45.7% and 34.3% respectively compared to a PCE of 100% at an S-D offset of 0.75 mm. Number of detector fibres were assigned as 2 fibres at 1.57 mm, 3 fibres at 2.68 mm and 4 fibres at 3.5 mm to obtain equivalent PCE and signal-to-noise ratio (SNR) between these three S-D offsets, i.e. 2 fibres × 68.6% PCE ≈3 fibres × 45.7% PCE ≈ 4 fibres × 34.3% PCE. This led to a design of 7 mm diameter probe that enclosed 36 detector fibres (100 micron in diameter) organised into 4 quadrants (Q1–4), with each quadrant containing 2, 3 and 4 fibres for the 3 ring of detectors – R1, R2 and R3 at the determined S-D offsets respectively (see Fig. 2B and C), as described earlier. The 36 detector fibres in the new probe design are aligned into a single line at the spectrograph input. The previous probe developed by Keller *et al*.^37^ was a single quadrant with four S-D offsets at 0.5, 1.5, 2.5 and 3.5 mm, while the currently designed probe incorporates three S-D offsets (1.57, 2.68 and 3.5 mm), but is radially symmetric in all 4 quadrants preventing the problem of directionality of signal collection (see Fig. 2B). Due to the increased number of detector fibres (36 in new probe vs 10 in the probe designed by Keller *et al*.), spectra with the same SNR can be obtained ~4 times faster. Furthermore, Raman signal is collected from a larger surface area of 38.5 mm^2^ with this probe compared to 19.6 mm^2^ area covered by the previous probe. The new probe would therefore require fewer points to cover the entire specimen surface, thus reducing the number of measurements needed to cover sample surface. For margin classification, Keller *et al*. had earlier established that an S-D offset of 3.5 mm could detect 1 mm thin tumours at a depth of 2 mm from the surface due to a relative spectral contribution of 5% from the tumour at that depth in the overall SORS spectra^34,35^. Since the current probe also has the same S-D offset of 3.5 mm, the relative spectral contribution threshold is set at 5% to classify margins based on signal acquired down to a depth of 2 mm for breast specimens. It must be noted that 12 Raman spectra are acquired at each measurement point – each spectrum corresponds to a ring of detectors (R1/R2/R3) in each probe quadrant (Fig. 2B) that could provide depth-resolved information for the purpose of SORS. Nonetheless instead of applying SORS, we notably reduced the duration of spectral analysis by simply averaging these 12 Raman spectra and obtaining the depth-averaged Raman spectra per measurement point for a quicker margin scan.

### Mechanism and workflow of the device

**Figure 3:**
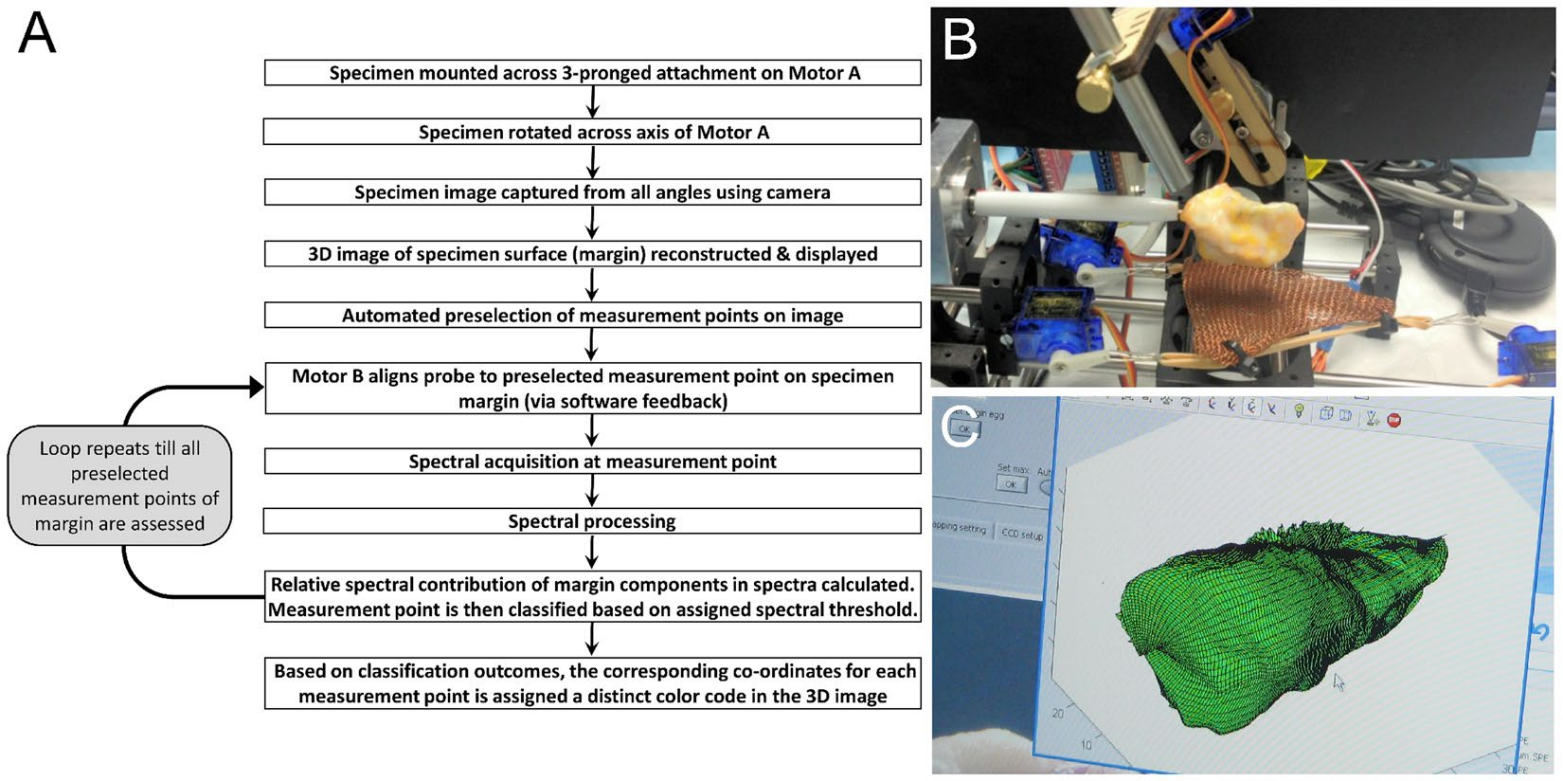
Mechanism of the automated 3D margin scanner. (**A**) Work-flow involved for margin classification with the prototype scanner. (**B**) Automated spectral acquisition from sample margins with the device. (**C**) Marginal composition being represented on a morphologically reconstructed image of breast specimen assessed with the designed scanner.

When the specimen is mounted onto the scanner using a 3-pronged attachment on Motor A, the surgical orientation of the specimen is marked using the software. The workflow of the system from specimen mounting to the eventual margin composition representation in 3D is summarised in Fig. 3A. As the specimen rotates, the integrated camera in front of the scanner (see Fig. 1A and B) captures specimen photographs at different angles to reconstruct a 3D morphological image of the specimen surface. From the reconstructed 3D image, coordinates for various measurement points are calculated and selected by the software to ensure evaluation of the entire sample surface. The distance between each measurement points on the margin and thus the total number of measurement points per specimen can be customized as per the user’s requirements. Measurements are then acquired at each of those points in an automated manner (see Fig. 3B). After all measurements are taken, the Raman spectra are calibrated, noise smoothed and fluorescence background subtracted as previously described^37,39^. Based on the spectrum recorded at each location, the classification module then categorises each measurement point on the margin. The final margin result is co-registered with the 3D reconstructed morphological image and displayed with the surgical orientation as depicted in Fig. 3C.

### Validation on margins of phantom sample

**Figure 4:**
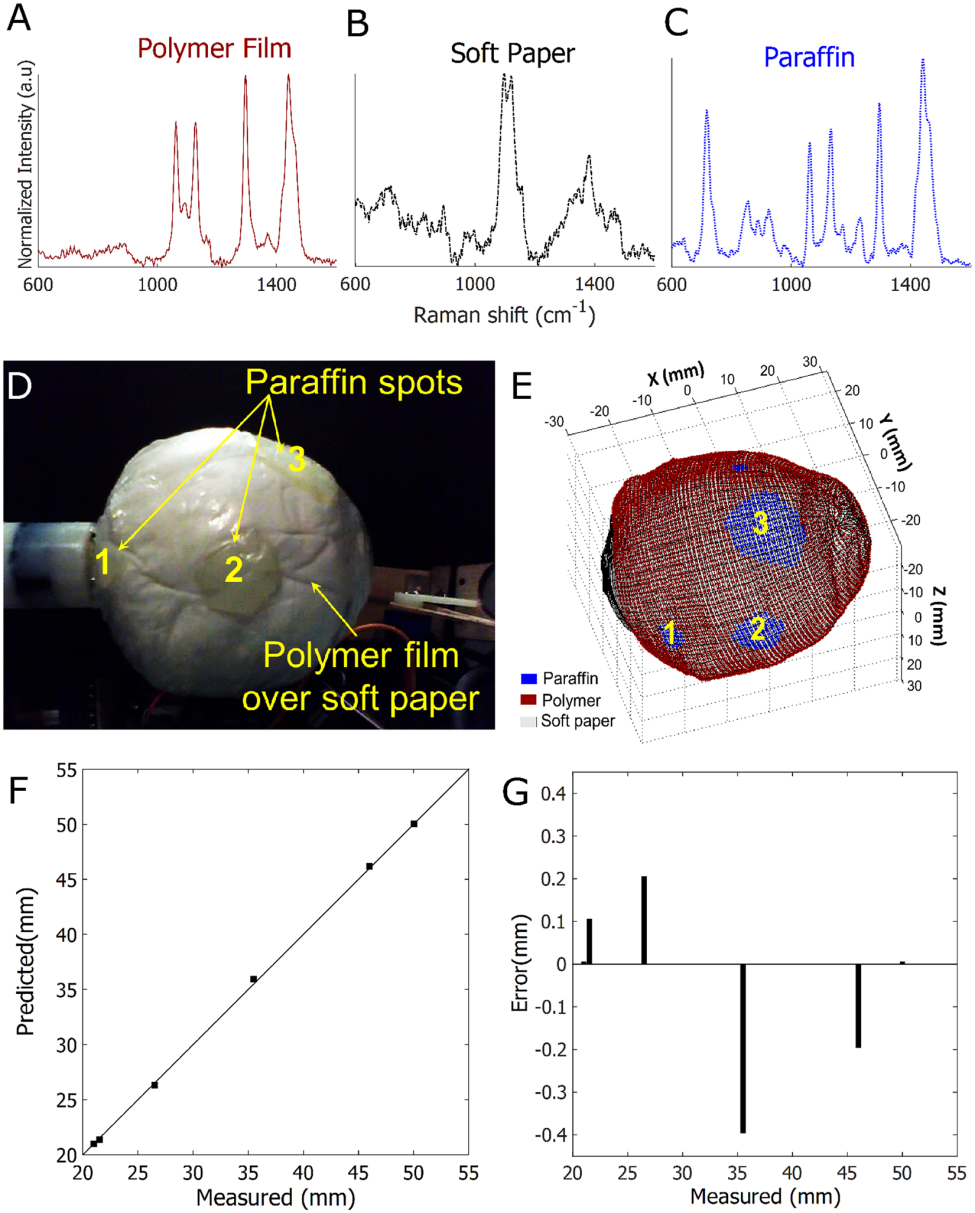
Testing on phantom sample with the automated 3D margin scanner. Raman spectra of (**A**) polymer film, (**B**) soft paper and (**C**) paraffin. (**D**) Spherical phantom sample (5 cm in diameter) composed of polymer film, soft paper and paraffin, (**E**) Automated 3D reconstruction of the phantom sample margin demonstrating spatial orientation of paraffin spots correlating well with the actual sample, (**F**) Plot showing correlation between the actual distance measured between the 4 paraffin spots and the predicted distance calculated from the reconstructed image (Coefficient of determination R^2^ = 0.99), (**G**) Graph displaying the absolute errors of predicted distance compared to the actual measured distances between the 4 paraffin spots, showing a maximal error <0.5 mm.

The phantom sample comprised of soft paper with embedded paraffin blocks wrapped in a 2 mm thin polymer film (Fig. 4D). Pure spectra of the three components constituting the phantom sample are depicted in Fig. 4A–C. Pure spectrum of each component was utilised in a classical least squares (CLS) model to calculate the relative contribution of polymer, soft paper and paraffin at individual points. In total, the CLS model was able to capture 97% of the cumulative variance of the dataset which confirmed the phantom sample composition with minimal contribution from other agents. Based on the automated image reconstruction and margin evaluation of the phantom sample, relative contributions of the soft paper, paraffin spots and polymer film in the sample margin are seen in Fig. 4E. Upon comparing Fig. 4D and E, the 4 spots classified as paraffin by the system co-registered precisely in location, size and shape with the actual paraffin spots in the phantom sample. To confirm spatial accuracy of the paraffin spots and 3D reconstruction of the phantom sample, the distance between the four spots predicted from the reconstructed 3D image, were then compared with the actual distance measured directly from the sample (Fig. 4G). Upon determining the absolute errors of predicted distance versus the true measured distance as displayed in Fig. 4F, it can be seen the 3D image of the sample surface/margin (See Supplementary Video 1) was reconstructed with an accurate localisation of the paraffin spots with an error < 0.5 mm. The findings obtained with the phantom sample thus validates the potential of this system to perform (i) automated 3D reconstruction of sample margins and (ii) precise margin evaluation with sensitivity to spatial orientation and chemical composition in the assessed sample.

### Margin analysis of excised breast specimens

**Figure 5:**
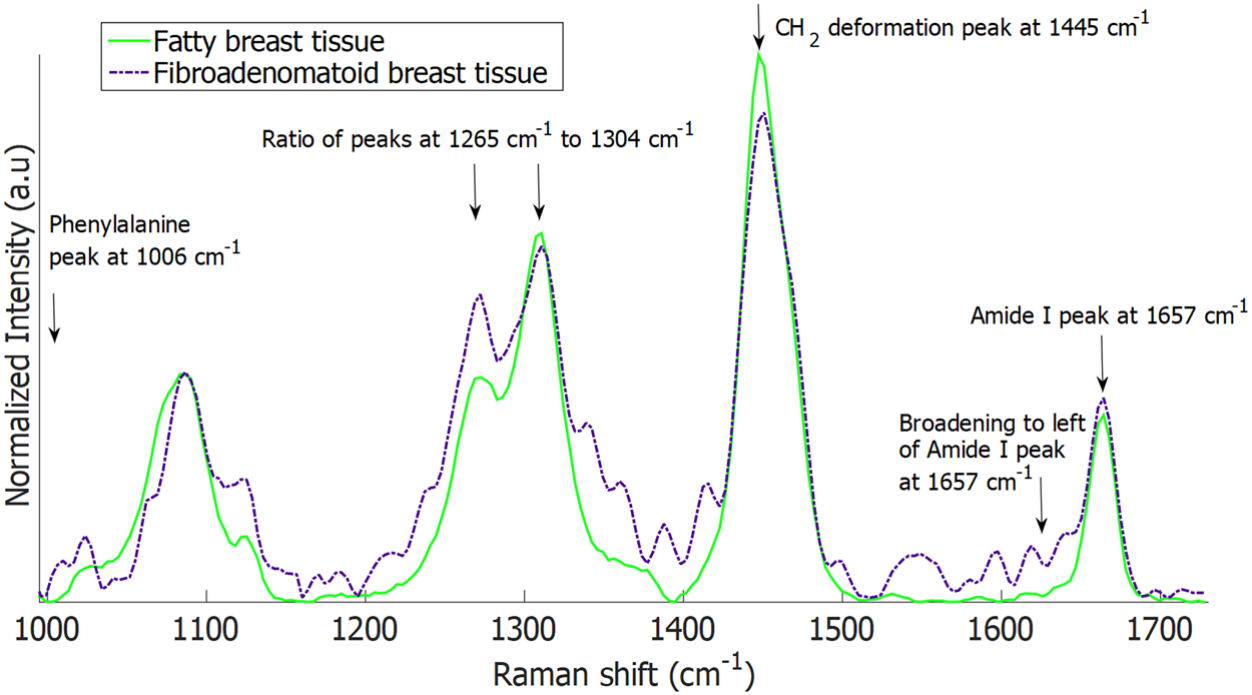
Raman spectra of normal and fibroadenomatoid regions in breast tissue used for the classical least squares (CLS) model for margin classification. Raman spectra obtained from fatty and fibroadenomatoid regions visually identified by the pathologist on mastectomy breast specimens. These pure Raman spectra were used for the classical least squares (CLS) model for margin classification. Compared to fatty breast tissue (continuous green line), Raman spectra of fibroadenomatoid breast tissue (blue dot-dash line) display (a) a notable phenylalanine peak at 1006 cm^−1^, (b) an increase in intensity at 1265 cm^−1^ relative to 1304 cm^−1^, (c) decreased intensity at 1445 cm^−1^ and left sided broadening of the peak at 1657 cm^−1^. The features indicate a relative increase in protein and decrease in lipid content in fibroadenomatoid breast compared to fatty breast.

Similar to the phantom sample, pure Raman spectra were first obtained from ‘fatty’ or ‘fibroadenomatoid’ regions to develop a CLS model for margin classification. This was achieved by performing single point-based Raman measurements on regions visually confirmed as ‘fatty’ or ‘fibroadenomatoid’ by the pathologists on the excised mastectomy (full breast) specimen. Characteristic features of pure Raman spectra that differed between fatty and fibroadenomatoid margins (Fig. 5) include (i) reduced intensity of CH_2_ deformation peak at 1445 cm^−1^, (ii) decreased relative ratio of 1304 to 1265 cm^−1^ peaks, (iii) presence of 1006 cm^−1^ peak attributed to phenylalanine and (iv) increased widening of the amide I peak around 1657 cm^−1^ which all denote increased protein and decreased lipid content in fibroadenomatoid margins relative to normal fatty tissue. Prior work have demonstrated similar spectral traits that differentiate between normal fatty breast tissue and fibroadenoma, indicating that these two tissue types have significantly different Raman spectra^28,30^.

**Figure 6:**
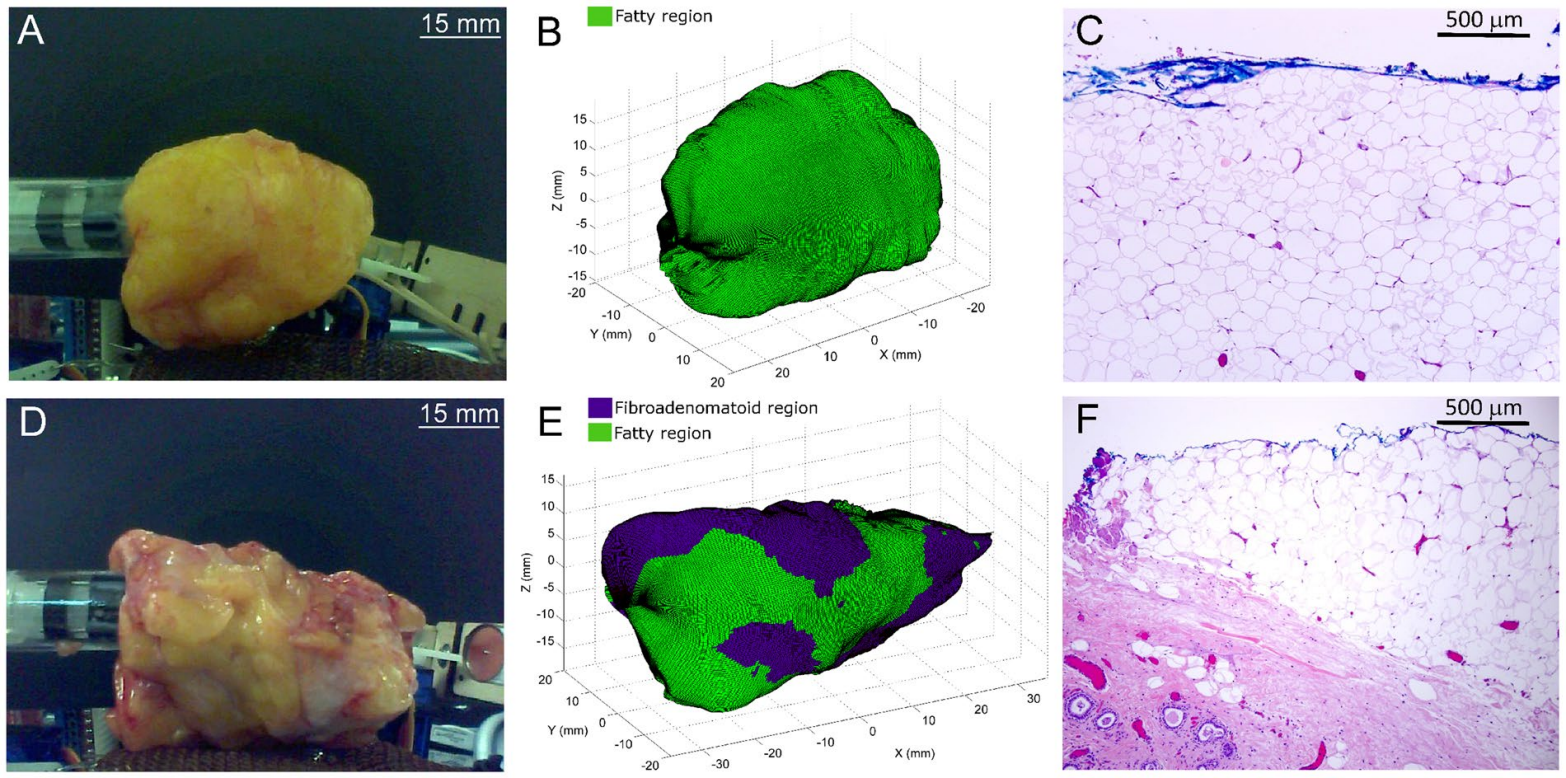
Automated 3D margin assessment of breast specimens. (**A**) Photograph of the breast specimen with fatty margins, (**B**) margins of the specimen rendered by the scanner and (**C**) 10X magnification of an H&E section from one of the blue inked biopsy spots in the fatty margin. (**D**),(**E**) and (**F**) depict the corresponding figures for a breast specimen with fibroadenomatoid margins. Note the increased fibro-epithelial composition at a depth of 0.5–1.5 mm from the margin in Fig. 6(F). (Colour code for margin classification in (**B**) and (**E**): green −>50% fat composition, blue −>50% fibroepithelial/fibro-glandular composition)

Subsequently, 3–6 cm wide breast tissues were cut out from the total mastectomy specimens to simulate ‘test lumpectomy’ samples for assessment with the 3D margin scanner. The total measurement time for the ‘cut out’ breast specimens (Dimension range: 3 × 1.5 × 1 cm to 6 × 2 × 2 cm) varied from 7–15 minutes. The aforementioned features of the pure Raman spectra (Fig. 5) obtained from pre-identified fatty and fibroadenomatoid regions, were then input into the CLS model. Using an assigned Raman spectral threshold, the developed CLS model analysed the relative spectral contributions at each measurement point on the margins of these test lumpectomy specimens and classified each point as fatty or fibroadenomatoid. As seen in Fig. 6A and D, the photographs of breast specimens with a fatty margin and a fibroadenomatoid margins are shown respectively. Figure 6B depicts a margin representation of the fatty margin specimen as scanned by this system. It can be seen that the entire margin for this specimen was assessed and classified as fatty (green) based on the detected Raman spectra. Histopathological evaluation of this specimen margins revealed predominantly adipocytes (fatty cells) with minimal stroma for depths of 1.5–2 mm from the blue inked surface margin (Fig. 6C). In contrast, the margin representation of the fibroadenomatoid specimen (Fig. 6B) by the scanner demonstrated several regions in the margins that were distinctly classified as fibroadenomatoid (blue) in the midst of fatty (green) zones (Fig. 6E). This was histopathologically confirmed (Fig. 6F), where extensive presence of fibroepithelial and/or fibro-glandular structures suggestive of fibroadenomatoid changes located at depths ranging from 0.5–1.5 mm can be seen from the blue inked margins of the surface of the specimen.

**Figure 7:**
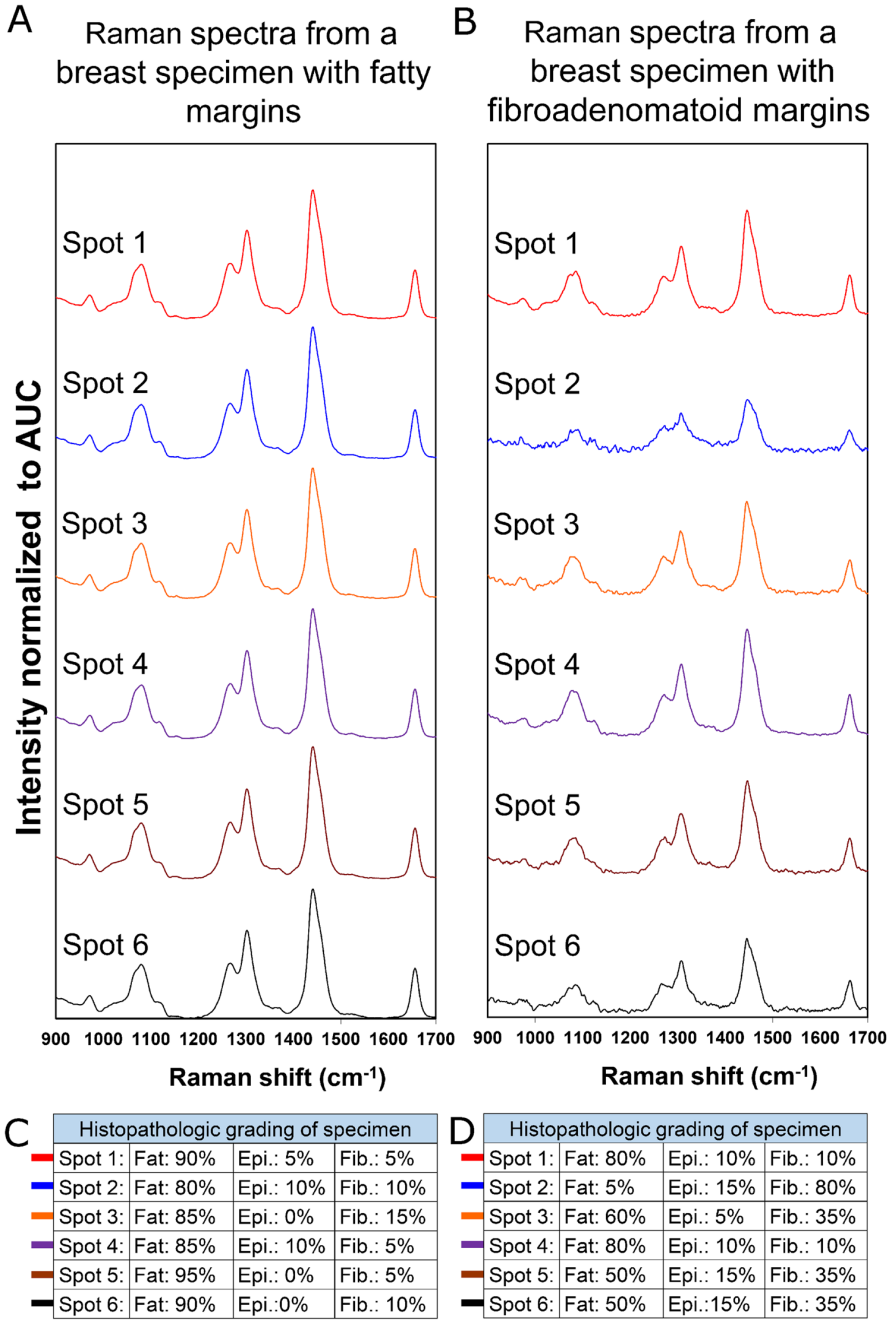
Comparison of depth-averaged Raman spectra and histopathological grading of the additional spots on margin assessed by the prototype scanner. Raman spectra with intensity normalised to area under curve (AUC) acquired by manual point measurements on margin. Spectra were obtained from 6 additional spots on fatty (**A**) and fibroadenomatoid margins (**B**). Histopathological grading and validation are indicated in the tables below (**C** and **D**) that display the percentage of fat, epithelial (Epi.), fibrous (Fib.) composition of the assessed margin spots. Note (i) decreased intensity at 1445 cm^−1^, (ii) the increased intensity of 1265 cm^−1^ relative to 1304 cm^−1^ and (iii) lower SNR of spot 2 in breast specimen with fibroadenomatoid margin (**B**) compared to fatty margin (**A**), which is validated by histopathological grading tabulated below.

For histopathological validation, Raman spectra were obtained from 4–6 additional spots in each specimen (a total of 28 spots over 5 specimens), which were inked after measurement and biopsied. Thirteen spots were categorised as fatty (≥50% fat composition) with the remaining fifteen as fibroadenomatoid (< 50% fat composition) based on histopathology. Spectra for all 6 spots (Fig. 7A) in a fatty margin specimen were similar and demonstrated strong peaks at 1304 and 1445 cm^−1^ corresponding to lipids, suggesting a homogeneous distribution of fatty tissue across the assessed margins, which was histopathologically confirmed (Fig. 7C). In comparison, Raman spectra from 6 corresponding spots from a specimen with fibroadenomatoid regions (Fig. 7B) varied highly. While the spectra for Spot 1 and Spot 4 displayed strong peaks at 1304 and 1445 cm^−1^, spectra for the remaining spots demonstrated (i) relatively reduced peak intensity at 1304 and 1445 cm^−1^, (ii) increased intensity of 1265 cm^−1^ relative to 1304 cm^−1^ and (iii) lower signal-to-noise ratio. This was indicative of a heterogeneous distribution of fatty tissue, interspersed with definite regions that exhibited reduced lipid and increased protein related peaks with noisier spectra. Histopathologic assessment (Fig. 7D) revealed that Spot 2 possessed the lowest fatty tissue composition at 5% when compared to fibrotic and glandular tissue that constituted 95%, which correlated well with the spectral findings where Spot 2 had the lowest intensity at 1304 and 1445 cm^−1^ peaks related to lipids. In contrast, Spot 1 and 4 that had the strongest intensity at these Raman peaks, were validated having the highest fatty tissue composition at 80% based on histopathologic evaluation.

**Figure 8:**
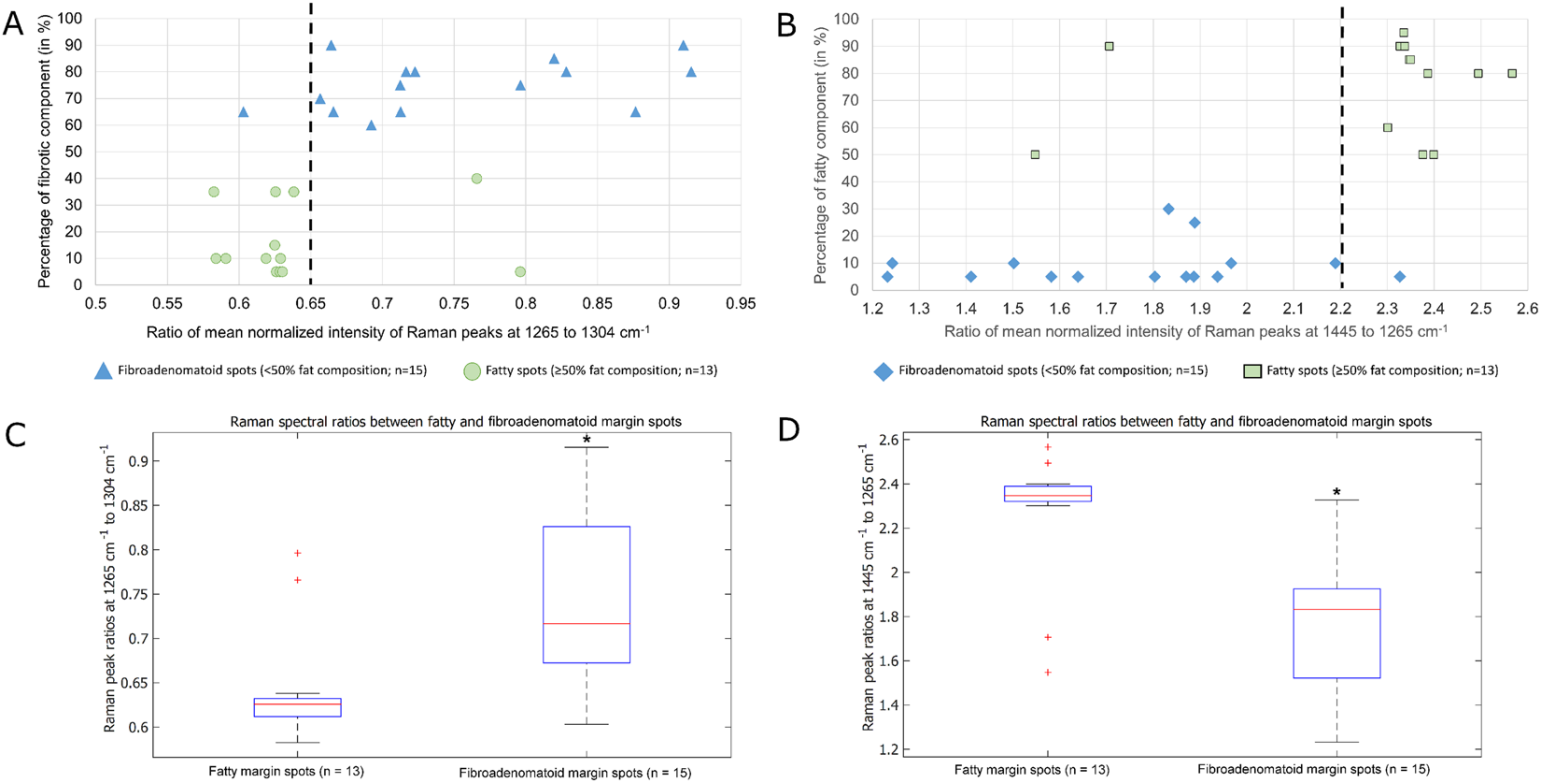
Correlation between Raman spectral ratios obtained with the automated scanner and histopathological grading. Raman spectral ratios obtained with the device at 1265 to 1304 cm^−1^ is significantly higher for spots classified histopathologically as fibroadenomatoid (blue triangle) when compared to those that were fatty (green circle) as seen in **A** and **C**, suggestive of higher protein composition in the former (2-tailed t-test of unequal variance, p = 0.0015). In contrast, Raman spectral ratio at 1445 to 1265 cm^−1^ is considerably lower for the fibroadenomatoid spots (blue diamond) than the fatty ones (green square) as observed in **B** and **D**, indicating a relatively higher lipid content in the fatty spots (2-tailed t-test of unequal variance, p = 0.00015). (*indicates a p-level < 0.01 for statistical significant difference between fibroadenomatoid and fatty spots from the evaluated breast specimens).

Raman spectral ratios evaluated at 1265 to 1304 cm^−1^ were found to be notably higher for the fibroadenomatoid spots (p = 0.0015), when compared with fatty spots (Fig. 8A and C). In contrast, the spectral ratio of 1445 cm^−1^ to 1265 cm^−1^ was significantly decreased in fibroadenomatoid spots (p = 0.00015), when compared with the assessed fatty spots (Fig. 8B and D). Raman spectra and histopathological grading for the 28 biopsied spots from all 5 breast specimens can be observed in Supplementary Figure 1. Inputting the spectra of these 28 spots into a machine-learning multivariate classification algorithm using sparse multinomial logistic regression (SMLR)^40^, yield a performance of 93% sensitivity (14/15 of fibroadenomatoid) and 85% specificity (11/13 of fatty) for an overall accuracy of 89% (kappa = 0.78) when correlated with histopathological grading.

## Discussion

This manuscript showcases the design and testing of an automated scanner prototype – Marginbot – for assessing breast margin composition of a specimen and providing a 3D representation of the same in relation to gross specimen morphology. The described device can evaluate margins in breast specimens as wide as 3–6 cm in 7–15 minutes. Testing in phantom samples indicate that this device could provide a 3D representation of the margin composition precisely with an absolute spatial error < 0.5 mm (Fig. 4D–G). More importantly, testing on excised breast specimens demonstrates that the designed device is sensitive to tissue composition in breast margins. Depth-averaged Raman spectra acquired from additional spots on specimen margins with this system, discriminated fibroadenomatoid from fatty areas with 93% sensitivity and 85% specificity when correlated with histopathological grading. While the 3D margin scanner has demonstrated feasibility for effectively distinguishing between fatty and fibroadenomatoid margins, the potential of this system is yet to be investigated for assessing margins of true lumpectomy specimens obtained during BCS of invasive carcinomas or DCIS. The device requires further development for optimal differentiation between tumour positive and negative margins, by augmenting certain features of the instrument. The margin classification algorithm utilized for this study predominantly relied on prominent Raman peaks at 1265, 1304, 1445 and 1657 cm^−1^ that served as relative indicators for lipid and protein levels in specimen margins to simply distinguish between fatty and fibroadenomatoid margins^37,41^. A more robust margin classification algorithm needs to be implemented for differentiating between tumour positive and negative margins amidst normal fatty or fibro-glandular breast tissue. Therefore, the algorithm should ideally involve a more complete decomposition of Raman spectra to measure contributions of other biochemical components like cell nuclei (DNA), cholesterol and collagen^28,31^. Furthermore, the relative spectral contribution threshold for this system is set presently to distinguish between a fatty and fibroadenomatoid margin. This threshold would need to be optimized to sensitively identify tumour positive margins during BCS. Regardless, we demonstrate the capability of an automated scanner to perform 3D margin evaluation for breast margins *ex vivo*. Prior work by Keller *et al*. has demonstrated that SORS can discriminate tumour margins *ex vivo* with a 95% sensitivity and 100% specificity using a manual point-based approach of spectral acquisition^37^. Therefore this scanner prototype in conjunction with a probe designed for SORS can perform automated complete margin scans, while holding the potential to simultaneously discriminate tumour margins in true lumpectomy specimens with high accuracy.

Our results demonstrate feasibility of a newly designed 3D scanner using depth-averaged Raman Spectroscopy (RS) in differentiating between fatty and fibroadenomatoid margins of breast tissues with intraoperative potential (Fig. 6B and E). Twenty five of the 28 additional margin spots tested for histopathological validation were correctly classified upon analysing spectral ratios at 1265/1304 cm^−1^ and 1441/1265 cm^−1^ (Fig. 8A–B) suggesting increased protein and reduced lipid content in the fibroadenomatoid as compared to fatty regions. This finding was validated with multivariate analysis of depth-averaged RS spectra that correctly classified the same 25 out of 28 spots. It is possible that for those 3 misclassified spots, RS spectra may have been acquired from deeper tissue structures, while the corresponding biopsy for histopathology may not have been as deep. Another feasible explanation for misclassification could be low SNR that could be improved by optimizing spectral acquisition parameters. A more robust histopathological validation would involve multiple biopsies at frequent intervals along probe measurement sites or detailed histopathological analysis of the entire specimen surface. However no more than 6 biopsies could be performed per specimen due to specimen size constraints. It must also be duly noted that extrapolating system performance from a few select histopathologically validated points to the entire specimen margins is a challenging concept due to unknown heterogeneity of the entire specimen margins. In this study 14/15 fibroadenomatoid spots and 11/13 fatty spots were correctly classified as validated by histology. All other areas of the specimen were not histologically evaluated for accurate estimation of system performance. Nonetheless if the performance accuracy for specimens as a whole were to be considered, 3 out of 4 specimens had all fibroadenomatoid zones correctly identified (75% sensitivity), while 2 out of 4 specimens had all fatty zones accurately classified (50% specificity). However, it must be borne in mind that a lowered performance accuracy should be expected with a small sample size of just 5 specimens. From a clinical point of view, a more cautious approach could be adopted by the device where one positive spot measurement should indicate suspicion of a positive margin. While the device would provide a ‘stringent outlook’ for tumour margin discrimination, the final decision regarding margin width would rest with the surgeon.

The automated 3D margin scanner achieved complete margin assessment within a clinically feasible duration of 7–15 minutes for the tested breast specimens. To provide perspective, frozen section biopsies which is commonly used for intraoperative breast margin evaluation, is associated with a turn-around time of ~20 minutes for just one biopsy. A pathologist when aided by an assistant that cuts sections for him/her, can probably evaluate about 2 to 4 (or more) frozen sections in 20 min, depending on complexity of the diagnosis and adequacy of tissue sampling. Nonetheless, this makes the process labor-intensive and still not provide information regarding the entire margins. In addition, frozen section biopsies is associated with errors due to inadequate margin sampling and freezing artifacts in the section^42,43^. In contrast, the prototype device can scan margins of a 6 cm wide specimen within 15 minutes. It must be noted that an oblong specimen, similar to the one obtained in our study, measuring 6 cm × 2 cm × 2 cm (surface area ~5600 mm^2^) typically requires ~145 data point acquisitions with a 7 mm diameter probe (contact area = 38.5 mm^2^) for entire surface coverage. However, the current system acquired 305 data points from the specimen thereby implying oversampling of the margin surface, which can be minimised to further reduce the scan time. Furthermore, breast tumours considered for lumpectomies/BCS are typically T1-T2 stage breast cancers with diameters of 5 cm or less^44^. The designed 7 mm diameter probe therefore would provide full coverage for a 5 cm diameter lumpectomy specimen with ~204 data points in 10.5 minutes. To further shorten BCS duration, various strategies may be considered towards decreasing the scanning procedure to under 10 minutes without affecting sensitivity of the device. Scan time can be potentially reduced by decreasing measurement points required per specimen. Another point to be considered is that the actual data acquisition step takes just 1 second/spot or ~3.5 minutes for complete surface coverage for a 5 cm diameter specimen. The remaining 7 minutes would be the time taken for translocating the probe from one spot to the next (Fig. 3A) over 204 spots. Optimising the probe translocation time with improved instrument design or potentially using multiple optical probes per measurement site can achieve quicker margin coverage and reduce the tumour margin assessment duration. By doing so, the performance of the automated 3D scanner can be enhanced to notably shorten OR times for BCS procedures, minimise incomplete margin evaluation and ensure complete tumour resection. While this device is being further optimized and tested for eventual intraoperative use, margin inking and subsequent histopathology remain the gold standard for margin status confirmation. As a result, intraoperative testing of the 3D margin scanner in a current scenario could potentially delay specimen margin inking by about 7–15 minutes for lumpectomies as wide as 4–6 cm. For complying with standard pathology protocols, future studies will involve specimen orientation and boundaries being marked with sutures for identification immediately upon excision. Since the specimen can be co-registered to real-space using the 3D scanner system, the specimen will be returned in its original orientation. Following the margin scan, inking can proceed immediately thereafter in accordance with the established pathology guidelines without affecting specimen integrity.

The margin scan duration is also influenced by the timeframe for analysing and classifying the acquired spectra at each measurement-point. It must be reiterated here that 12 spectra are acquired at each measurement point, where each spectrum corresponds to a ring of detectors (R1/R2/R3) in each probe quadrant, e.g. Spectrum #1 is acquired from the 2 detector fibres in R1 of the upper right quadrant (Q1) in the probe (Fig. 2B). Each of these 12 spectra provide spatial and depth resolved tissue information from the measurement point, as demonstrated earlier to be a distinct ability of SORS^33,34,37^. For achieving a clinically feasible margin scan time, we shortened the duration required for spectral analysis, by simply averaging the 12 spectra and obtaining the depth-averaged Raman spectra per measurement point instead of performing SORS. Implementing a more comprehensive data processing algorithm can aid in rapid spectral analysis from each detector rings (R1-R3) independently to track tumour positive margins in a depth resolved manner and aid in exploiting the potential of SORS using the described automated scanner.

Loss of breast tissue firmness over time can prove challenging during margin assessment, as consistent specimen orientation and firm probe-tissue contact is essential for an accurate 3D margin representation. Brown *et al*. utilised a plexiform glass enclosure that optimally stabilised the specimen with its six walls^19^, but this approach limits the flexibility to evaluate specimens of varying size and shape. In contrast, our instrument design adopts a non-enclosure approach that allows the probe to move in 3D over the specimen (see Figs 1B and 3C) which ensures flexibility for specimen evaluation. While contact measurements with the optical probe are non-invasive and does not compromise specimen integrity, this current scanner design is limited with the specimen requiring to be pinned to the rotation motor at a 7 mm wide spot. To eliminate this blind spot, an additional point-based spectral measurement was obtained from this region using the 7 mm diameter probe after the automated margin scan. Although specimen pinning at this single spot pose only a minimal risk to specimen margin integrity, the specimen holder of the scanner can be redesigned to potentially eliminate the need to pin the sample along the rotating motor for minimizing specimen interference. A newer design for the specimen holder could additionally aim to support the specimen and concurrently stabilize its centre of gravity to allow margin assessment of specimens of varied sizes and shapes. The 3D margin scanner can be further optimized by implementing higher degree of freedom for probe movement over the specimen.

Various optical techniques, such as ESS^18^, DRS^19–21^, OCT^22^, PAT^23^ and Raman spectroscopy^31^ have been investigated for their potential to perform intraoperative breast tumour margin assessment with varying degree of success. For eventual clinical feasibility, the modality should ideally aim to sufficiently achieve (i) rapid margin coverage for entire specimen, (ii) provide depth-resolved information about the margins and (iii) have high accuracy in discriminating between tumour negative and positive margins. The distinct ability to obtain spectral information at subcellular levels makes ESS a novel tool for margin evaluation. However ESS is limited by lengthier scan times requiring about 1 second per 0.15 mm^2^, with a performance sensitivity below 70%^18^. A more optimal performance for scanning breast margins in entirety was demonstrated using DRS with 79.8% sensitivity and 66.7% specificity. The modality can measure volumetric signal from tissues down to a depth of 3 mm at 0.75 mm resolution over 10.28 cm^2^ in 13.1 minutes^19–21,45^. Although DRS can perform quick margin coverage and achieve optimum depth sampling, it scores lower in specificity and lacks the ability to provide depth resolved information. In comparison, OCT and PAT can attain rapid margin coverage and also provide depth resolved information, but with only 82% and 80% specificity respectively^22,23^. While Raman spectroscopy performs with 94% sensitivity and 96% specificity in assessing breast tumour margins^28^, it suffers from relatively weak signals and not being capable of obtaining tissue volumetric information in its conventional setting. Subsequently more researchers are leaning towards multi-modal imaging approaches to achieve all the three criteria and develop a viable intraoperative tool for breast tumour margin evaluation. While our current approach relied on applying depth-averaged RS for margin classification, the described automated 3D scanner could be used with the probe potentially acquiring depth-resolved Raman spectra as in SORS, for optimal performance accuracy and depth resolution^34,35,37^. Our current system requires a scan time of 15 minutes with 305 data points over a breast specimen with surface area of ~56 cm^2^ (6 cm × 2 cm × 2 cm), at a spatial interval of 3.5 mm between each measurement points. The precision of spectral mapping of margins can be improved further by decreasing the spatial intervals between measurement points to as low as 0.5 mm (as performed for the phantom sample – Fig. 4E), but at the cost of a lengthier margin scan duration due to additional measurement points. The probe used in this system has an S-D offset of 3.5 mm that can acquire depth resolved information from layers as deep as 2 mm from surface^34^ which is beneficial for ensuring an adequate tumour negative margin during BCS^10^. The described system yielded a 93% sensitivity and 85% specificity as validated on select biopsy spots from the specimens, and additionally provides the margin composition in relation to anatomical coordinates of the specimen in 3D. The current design of the 3D scanner is also expedient in being a flexible unit that can accommodate other probe-based optical techniques such as OCT, DRS or fluorescence based imaging for the purpose of multi-modal margin evaluation in 3D during tumour excision surgeries, if required.

In summary, we present a device that performs automated margin scanning with the ability to overlay spectral information of the scanned margins onto a 3D reconstructed image of the specimen surface. Since point-based SORS measurements has already demonstrated a high accuracy in tumour margin discrimination^37^, the ability of a automated margin scanning system in conjunction with SORS-based spectral acquisition can be exploited further to differentiate between tumour negative and positive margins during BCS. 3D representation of the tumour margin composition provided by the device would be extremely valuable in providing the surgeon with anatomical coordinates for regions requiring surgical re-excision *in-situ* during BCS. Moreover, the scanner can be potentially used for automated tumour margin evaluation for other organ cancers that require tissue conserving surgeries like brain, prostate, kidney and soft tissue cancers. The findings described in our study indicate that the designed automated 3D scanner holds immense potential for real-time surgical guidance for tumour margin evaluation during BCS.

## Materials and Methods

### Instrumentation

A 7 mm diameter optical probe (EMVision, Loxahatchee, Florida) is used to deliver 80 mW of power from a 785 nm diode laser (Innovative Photonics Solutions, Monmouth Junction, New Jersey). Raman signal is acquired by detector fibres in the probe and delivered to a near-infrared-optimised spectrograph (LS785, Princeton Instruments, Princeton, New Jersey), and recorded by a deep depletion, thermo-electrically cooled CCD (Pixis 400BR, Princeton Instruments). The 3D scanner component of Marginbot consists of two motors and servomotors (Tower Pro, Shenzen City, China), which is controlled by a laptop. A customised program code written in LabVIEW (National Instruments, Austin, Texas) and MATLAB (Mathworks, Natick, Massachusetts) can (i) reconstruct a 3D diagram of the specimen margin based from captured images of it from all angles by a camera (Genius, Doral, Florida), (ii) control the movement of motors and servomotors, and (iii) receive feedback from these components via a signal relay port (Belkins, Playa Vista, California).

### Data analysis for margin evaluation

The optical probe records 12 Raman spectra corresponding to three rings/quadrant for four quadrants at each point that was averaged for each spot over a sampling depth of 2 mm and a spot size of 7 mm diameter in this study. Neon-argon lamp, naphthalene, and acetaminophen were used to calibrate the wavenumber axis for the acquired Raman spectra, and a National Instrument Standards and Technology-calibrated tungsten-halogen lamp (Oriel Instruments, Irvine, California) was utilised to correct for wavelength response of the system. A classical least squares (CLS) model for calculating the relative spectral composition of acquired spectra, was generated using PLS_Toolbox (Version 7.5, Eigenvector Research, Wenatchee, Washington) with MATLAB (Mathworks, Natick, Massachusetts) software. The acquired Raman spectrum was first noise smoothed and fluorescence background subtracted as described in earlier studies^37,39^, following which it was normalised to its area under curve (AUC) for each specimen to minimise inter-specimen variation in Raman signal intensity.

### Phantom sample testing

A 5 cm diameter spherical phantom was made to imitate excised breast tumour specimens with soft paper, polymer film and paraffin. Four paraffin spots (dimensions: 3 × 3 mm – 12 × 12 mm) intended to mimic tumour positive margins were embedded onto a ball of soft paper and were covered with a 2 mm thick layer of polymer film. Pure spectra were obtained for the individual phantom sample components. For margin assessment, the sample was automatically measured with a step of 0.5 mm. The measurement time was 0.5 second per measurements and a total of 8666 data points were collected from the entire surface. Prior to scanning, distances between all 4 paraffin spots were measured on the phantom. Results were then later correlated with the distances between representative paraffin spots in the reconstructed morphologic image of the phantom.

### Breast tissue from prophylactic mastectomies

This study was conducted in accordance with the Declaration of Helsinki and its amendments and was approved by the Vanderbilt Institutional Review Board (IRB) - #050551 at Vanderbilt University, Nashville, Tennessee, USA and the U.S. Army Medical Research and Material Command’s Human Research Protection Office at Fort Detrick, Maryland, USA Specimens were obtained from 5 patients undergoing prophylactic total mastectomy who signed a written informed consent prior to surgery for recruitment in this study and allowing their mastectomy specimens to be used for research purposes. Since these were prophylactic mastectomy procedures, no margin inking was required for the excised breast tissue as per surgical guidelines. During prophylactic mastectomy, the entire breast was excised from the patient and pure Raman spectra were obtained using single point-based SORS measurements from regions on the complete breast tissue, identified as ‘fatty’ or ‘fibroadenomatoid’ by the pathologists using gross examination. The spectra thus obtained (Fig. 5) were input for the CLS model that would be utilized for the margin classification algorithm at a later stage. Following this step, samples that measured 3–6 cm in width were surgically cut from the total breast specimen to simulate a lumpectomy specimen, and were then evaluated with the 3D margin scanner and had the margins classified using the aforementioned margin classification algorithm. Measurement steps were taken at 3.5 mm with the measurement time per spot was 1 second. The total measurement time for the ‘cut out’ breast specimens (3 × 1.5 × 1 cm to 6 × 2 × 2 cm) ranged from 7–15 minutes. 50–305 data points were utilised to cover entire specimen surface depending on the specimen size. Single point measurements were performed on 4–6 additional spots on the specimen surface, which were inked after measurement, biopsied and fixed for histopathologic validation. Biopsies were serially sectioned, stained and evaluated by the pathologists who graded the percentage of fatty tissue, epithelial and fibrotic components per slide section semi-quantitatively for spectral correlation. Margin biopsies with less than 50% were considered fibroadenomatoid, while the remaining were termed fatty.

Raman spectral ratios from the biopsied spots at (i) 1265 to 1304 cm^−1^ and (ii) 1445 to 1265 cm^−1^ were compared between fatty and fibroadenomatoid margin spots to determine significant differences, if any, using a 2-tailed student’s t-test assuming unequal variance, with a p-value < 0.01 being considered as statistically significant. Additionally, these spectra were classified using a SMLR toolbox (Duke University, Durham, North Carolina)^40^, that computed the posterior probability of Raman spectrum belonging to ‘fatty’ or ‘fibroadenomatoid’ class based on a training set. A leave-one-specimen-out cross validation approach was used for the SMLR algorithm where the data were categorised into ‘n’ subsets (a subset comprising of spectra from all biopsied spots in one breast specimen) with n = 5. The algorithm identifies spectral features in a training set and uses it for classifying an unlabeled subset that is held out. Sensitivity, specificity and overall accuracy of the input spectra was thus determined by correlating with histopathologic grading. Classification was achieved by using a Laplacian prior probability with direct kernel and a lambda value of 0.01 in the algorithm without adding a bias term.

### Data Availability

The data generated during this study are included in this paper along with supplementary data files. The complete dataset is available from the corresponding author on reasonable request.

## Electronic supplementary material

Supplementary Video 1

Supplementary Figure 1
