# Supplementary material for: Evaluating feasibility of an automated 3-dimensional scanner using Raman spectroscopy for intraoperative breast margin assessment
Source: Sci Rep. 2017 Oct 19;7:13548. doi: 10.1038/s41598-017-13237-y (PMC5648832; doi:10.1038/s41598-017-13237-y)
Supplement: Supplementary file 2 — Supplementary Figure 1 [file 41598_2017_13237_MOESM2_ESM.pdf]

Evaluating feasibility of an automated 3-dimensional scanner using Raman spectroscopy for intraoperative breast margin assessment

G. Thomas[1, 2†], T-Q. Nguyen[2, 3†], I. J. Pence[1, 2], B. Caldwell[1, 2], M. E. O'Connor[1, 2], J. Giltnane[4, 5], M. E. Sanders[5], A. Grau[6], I. Meszoely[6], M. Hooks[6], M. C. Kelley[6], A. Mahadevan-Jansen[1, 2*]

[1]Vanderbilt Biophotonics Center, Vanderbilt University, Nashville, TN 37235

[2]Department of Biomedical Engineering, Vanderbilt University, Nashville, TN 37235

[3]Department of Biomedical Engineering, Northwestern University, Evanston, IL 60208

[4]Genentech, San Francisco, CA 94080.

[5]Division of Pathology, Vanderbilt University Medical Center, Nashville, TN 37232

[6]Division of Surgical Oncology, Vanderbilt University Medical Center, Nashville, TN 37232

*Corresponding author: anita.mahadevan-jansen@vanderbilt.edu

†These authors contributed equally for first authorship.

Supplementary Material Information Guide:

Supplementary Video 1: A 3D scan of margins of a phantom sample mimicking a positive breast tumor margin. The video depicts a three dimensional (3D) view of the margins of a phantom sample that mimics a positive breast tumor margin. The scanner provides biochemical information about the entire margins of the phantom sample margin in 3D.

Supplementary Figure 1: Depth-averaged Raman spectra and corresponding histopathological grading of additional spots assessed by the prototype scanner for all 5 breast specimens. Of the 28 spots from 5 breast specimens assessed in this study by the scanner, 25 were

classified correctly when correlated with their corresponding histopathological grading. 2 fatty spots were misclassified as fibroadenomatoid, while one fibroadenomatoid spot was incorrectly categorised as fatty. A biopsied spot was considered fatty histopathologically if 50% or more of the tissue was composed of fat and fibroadenomatoid if the fat composition was less than 50%

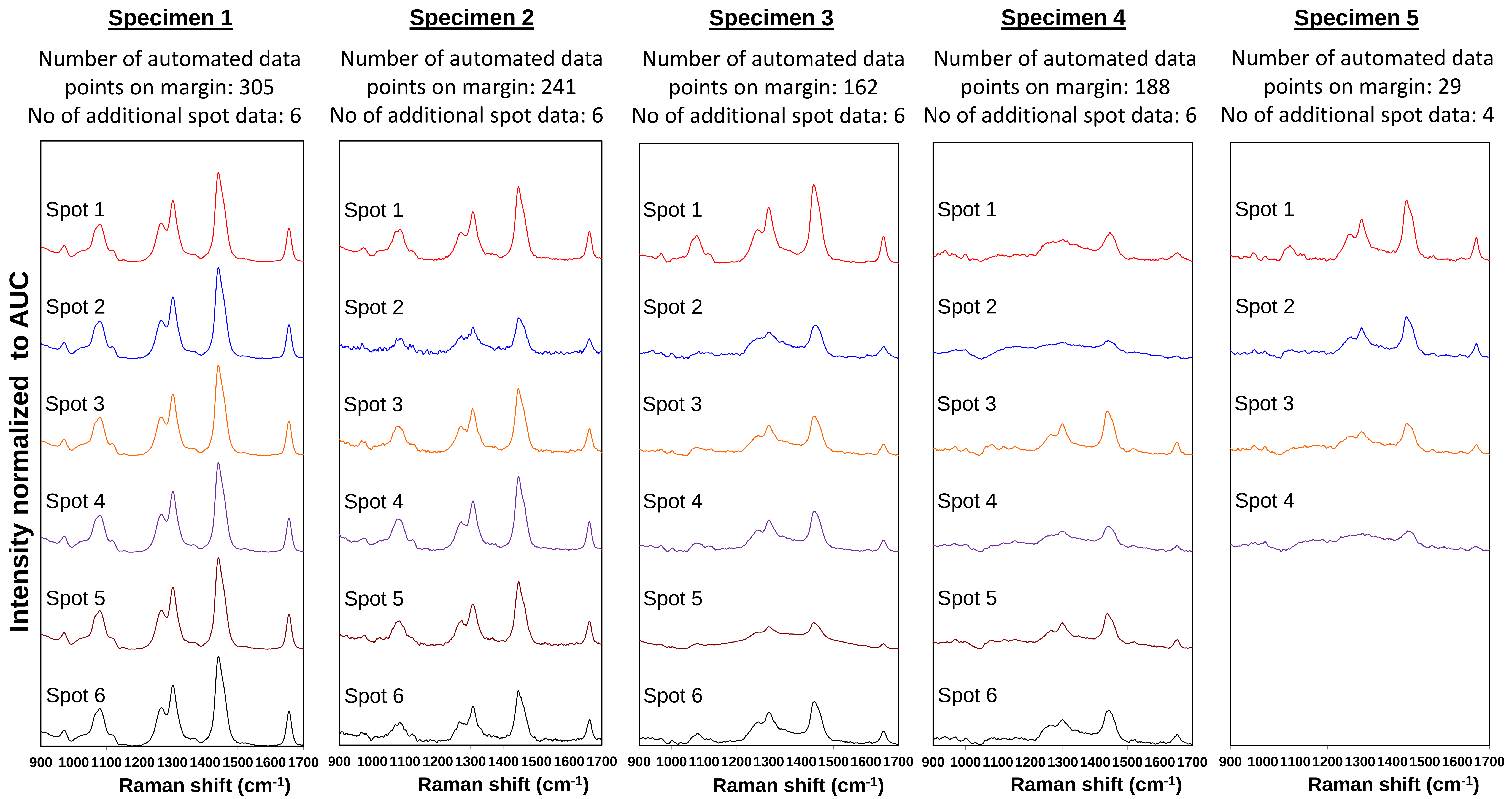

Specimen 1

Histopathologic grading of specimen		
Spot 1: Fat: 90%	Epi.: 5%	Fib.: 5%
Spot 2: Fat: 80%	Epi.: 10%	Fib.: 10%
Spot 3: Fat: 85%	Epi.: 0%	Fib.: 15%
Spot 4: Fat: 85%	Epi.: 10%	Fib.: 5%
Spot 5: Fat: 95%	Epi.: 0%	Fib.: 5%
Spot 6: Fat: 90%	Epi.:0%	Fib.: 10%

Specimen 2

Histopathologic grading of specimen		
Spot 1: Fat: 80%	Epi.: 10%	Fib.: 10%
Spot 2: Fat: 5%	Epi.: 15%	Fib.: 80%
Spot 3: Fat: 60%	Epi.: 5%	Fib.: 35%
Spot 4: Fat: 80%	Epi.: 10%	Fib.: 10%
Spot 5: Fat: 50%	Epi.: 15%	Fib.: 35%
Spot 6: Fat: 50%	Epi.:15%	Fib.: 35%

Specimen 3

Histopathologic grading of specimen		
Spot 1: Fat: 5%	Epi.: 30%	Fib.: 65%
Spot 2: Fat: 5%	Epi.: 20%	Fib.: 75%
Spot 3: Fat: 5%	Epi.: 5%	Fib.: 90%
Spot 4: Fat: 30%	Epi.: 10%	Fib.: 60%
Spot 5: Fat: 50%	Epi.: 10%	Fib.: 40%
Spot 6: Fat: 5%	Epi.:20%	Fib.: 75%

Specimen 4

Histopathologic grading of specimen		
Spot 1: Fat: 5%	Epi.: 5%	Fib.: 90%
Spot 2: Fat: 10%	Epi.: 25%	Fib.: 65%
Spot 3: Fat: 10%	Epi.: 25%	Fib.: 65%
Spot 4: Fat: 10%	Epi.: 10%	Fib.: 80%
Spot 5: Fat: 25%	Epi.: 10%	Fib.: 65%
Spot 6: Fat: 90%	Epi.:5%	Fib.: 5%

Specimen 5

Histopathologic grading of specimen		
Spot 1: Fat: 15%	Epi.: 20%	Fib.: 70%
Spot 2: Fat: 5%	Epi.: 15%	Fib.: 80%
Spot 3: Fat: 5%	Epi.: 10%	Fib.: 85%
Spot 4: Fat: 5%	Epi.: 15%	Fib.: 80%

Misclassified spots (3/28):

(1) Specimen 3, Spot 1 classified as Fatty; Histopathological diagnosis: Fibroadenomatoid

(2) Specimen 3, Spot 5 classified as Fibroadenomatoid; Histopathological diagnosis: Fatty

(3) Specimen 4, Spot 6 classified as Fibroadenomatoid; Histopathological diagnosis: Fatty

Supplementary Figure 1. Depth-averaged Raman spectra and corresponding histopathological grading of additional spots assessed by the prototype scanner for all 5 breast specimens. Of the 28 spots from 5 breast specimens assessed in this study by the scanner, 25 were classified correctly when correlated with their corresponding histopathological grading. 2 fatty spots were misclassified as fibroadenomatoid, while one fibroadenomatoid spot was incorrectly categorised as fatty. A biopsied spot was considered fatty histopathologically if 50% or more of the tissue was composed of fat and fibroadenomatoid if the fat composition was less than 50%
